# A randomized, placebo-controlled, double-blinded trial of MRSA throat carriage treatment, with either standard decolonization alone or in combination with oral clindamycin

**DOI:** 10.1186/s13063-022-06443-1

**Published:** 2022-06-16

**Authors:** Mona Katrine Alberthe Holm, Heidi Karin Meiniche, Michael Pedersen, Helle Brander Eriksen, Henrik Westh, Barbara J. Holzknecht, Mette Damkjær Bartels

**Affiliations:** 1 Department of Clinical Microbiology, Copenhagen University Hospital Amager and Hvidovre, Kettegaard alle 30, 2650 Hvidovre, Copenhagen, Denmark; 2 Department of Clinical Microbiology, Copenhagen University Hospital Herlev and Gentofte, Herlev, Denmark; 3 Department of Clinical Medicine, University of Copenhagen, Copenhagen, Denmark

**Keywords:** Methicillin-resistant *Staphylococcus aureus*, MRSA throat carriage, Treatment failure, MRSA decolonization, MRSA eradication

## Abstract

**Background:**

*Staphylococcus aureus* is a frequent colonizer of the human skin and mucous membranes but can also cause a variety of serious infections. Antimicrobial resistance is an increasing worldwide challenge and is mainly driven by an overuse of antimicrobials*.* To avoid the spread of methicillin-resistant *Staphylococcus aureus* (MRSA) in Denmark, the Danish Health Authority recommends decolonization treatment of MRSA carriers and their household contacts. Standard decolonization treatment includes chlorhexidine body wash and mupirocin nasal ointment, especially throat carriage is difficult to treat. The broad-spectrum antibiotic, clindamycin, is often added to the decolonization treatment, but there is currently low scientific evidence for this treatment.

**Aim:**

To investigate whether the addition of clindamycin to the standard decolonization treatment increases decolonization success in MRSA throat carriers.

**Methods:**

A randomized, placebo-controlled, double-blinded trial, including patients ≥ 18 years, who tested MRSA positive in the throat after completing one standard decolonization treatment. All carriers included in the trial receive standard decolonization treatment and are randomized to treatment with either placebo or clindamycin capsules for 10 days. We plan to include 40 participants in each of the two treatment arms.

**Discussion:**

Due to the lack of consistent scientific evidence of clindamycin’s effect in MRSA decolonization and the worldwide urgent need to reduce the use of antibiotics, we judged that a 30% increase in the decolonization success rate in carriers treated with clindamycin is appropriate to justify prescribing clindamycin as part of the decolonization treatment of asymptomatic MRSA carriers.

**Trial registration:**

EudraCT number 2019-002631-29

**Supplementary Information:**

The online version contains supplementary material available at 10.1186/s13063-022-06443-1.

## Administrative information

Note: The numbers in curly brackets in this protocol refer to SPIRIT checklist item numbers. The order of the items has been modified to group similar items (see http://www.equator-network.org/reporting-guidelines/spirit-2013-statement-defining-standard-protocol-items-for-clinical-trials/).

**Table:** 

Title {1}	A randomized, placebo-controlled, double-blinded trial of MRSA throat carriage treatment, with either standard decolonization alone or in combination with oral clindamycin
Trial registration {2a}.	The trial was registered on June 17, 2019 with number 2019-002631-29 [EU Clinical Trial Register]. NCT04104178 [ClinicalTrials.gov]
Protocol version {3}	Version 7, was approved by the Regional Research Ethical Committee on June 6, 2020, registered with number H-19062898.
Funding {4}	Amager and Hvidovre Hospital, the Hvidovre Hospital’s administration, Christian Larsen and Judge Ellen Larsens Fond, Lizzi and Mogens Staal Fonden, Else og Mogens Wedell-Wedellsborgs Fond, Thora and Viggo Groves Mindelegat, and Aase & Ejnar Danielsens Fond.
Author details {5a}	Mona Katrine Alberthe Holm^1^, Heidi Karin Meiniche^1^, Michael Pedersen^1^, Helle Brander Eriksen^2^, Henrik Westh^1,3^, Barbara J. Holzknecht^2,3^, Mette Damkjær Bartels^1,3^ ^1^Department of Clinical Microbiology, Copenhagen University Hospital - Amager and Hvidovre, Copenhagen, Denmark ^2^Department of Clinical Microbiology, Copenhagen University Hospital – Herlev and Gentofte, Herlev, Denmark ^3^Dept of Clinical Medicine, University of Copenhagen, Copenhagen, Denmark
Name and contact information for the trial sponsor {5b}	Sponsor: Mette Damkjær Bartels, Consultant, PhD /Head of MRSA Knowledge Center /Department of Clinical Microbiology 445 Amager and Hvidovre Hospital Kettegaard alle 30 DK-2650 Hvidovre Denmark mette.damkjaer.bartels@regionh.dk
Role of sponsor {5c}	Development of the idea and study design, conceptualization, supervision, data curation, access and acquisition, analysis and interpretation of the data and results, and critical revision of the protocol. The sponsor furthermore has the ultimate authority of reporting adverse and serious adverse events.

## Background and rationale {6a}

*Staphylococcus aureus* (*S. aureus*) frequently colonizes the human skin and mucous membranes. Longitudinal studies usually divide *S. aureus* carriers into either persistent or intermittent carriers, but because the number of samplings, follow-up periods, and study populations differ between studies, the designation of carrier state is inconsistent [1]. It has been estimated that around 20% of the population are persistent carriers, 60% are intermittent carriers, and 20% are non-carriers [2]. *S. aureus* is an opportunistic pathogen that can become invasive and cause a large spectrum of infections. *S. aureus* is frequently the cause of skin and soft tissue infections, e.g., wounds, furuncles, and abscesses, but can also cause arthritis, osteomyelitis, and endocarditis and is a common pathogen in surgical site infections and in infections related to foreign bodies, such as catheters and prosthetic devices [3].

Antimicrobial resistance is an increasing challenge worldwide. The first methicillin-resistant *Staphylococcus aureus* (MRSA) were seen in 1961 just 1 year after the antibiotic methicillin was introduced [4]. For several decades, MRSA was mainly hospital-associated, but since the mid-1990s, it has become increasingly prevalent in the community where it often affects children and younger adults [5, 6]. MRSA can cause the same types of infections as methicillin-susceptible *S. aureus*, but often only colonizes the skin and mucus membranes. MRSA carriers are at risk of developing infections [7] as well as to transmit MRSA to others. National guidelines on how to treat MRSA carriage are available in some countries, but the approach often differs between countries. The Danish Health Authority has published a national guideline on how to prevent the spread of MRSA to keep the prevalence of MRSA low in Denmark [8]. Laboratory findings of MRSA are routinely reported to the Danish Patient Safety Authority and the national reference laboratory, Statens Serum Institut, in order to monitor the MRSA prevalence in Denmark and identify outbreaks [9]. The guideline recommends that MRSA carriers and their household members undergo a 5-day decolonization treatment regime, consisting of mupirocin 2% nasal ointment twice daily in both nostrils, daily full body wash with chlorhexidine 4% soap, and performs thorough cleaning of the home. Unfortunately, many carriers are still MRSA-positive after completing the treatment, and especially throat carriers are difficult to treat [5]. In case of treatment failure, a new treatment is often recommended. If the patient is a throat carrier, we sometimes add antibiotics to the standard treatment on the third decolonization attempt, clindamycin being the first-line choice in our region, as we hypothesize that the treatment is more effective when clindamycin is added. In a recently published retrospective study by our group describing the MRSA treatment data of 164 patients from Copenhagen [5], we found that throat carriers had a higher treatment failure rate, but adding clindamycin to the first decolonization treatment did not significantly increase the success rate. However, looking at the second decolonization treatment, our data (not published) showed that 57% of the throat carriers treated with clindamycin became MRSA-free compared to 29% of the throat carriers not receiving clindamycin, indicating that there might be an effect of adding clindamycin to the second treatment. We find it likely that the non-significant effect following the first treatment was partly due to many patients being intermittent carriers, and thus, the outcome of the first treatment is highly influenced by the spontaneous loss of MRSA. We consider patients that are still throat carriers after one decolonization treatment to be more permanent carriers with a lower chance of spontaneous loss influencing the treatment outcome. As we now rarely use clindamycin for the first decolonization treatment, but often for the second treatment in throat carriers, we decided to design a randomized controlled trial (RCT) testing the effect of adding clindamycin to MRSA throat carriers for their second decolonization treatment. To our knowledge, the only RCT examining non-infected/non-surgical MRSA carriers including clindamycin for treatment of MRSA carriage is a Swedish study that showed a significant effect of a standard treatment plus the antibiotic rifampicin in combination with either clindamycin or sulfamethoxazole/trimethoprim compared to the standard treatment alone in long-term MRSA carriers [10]. As most of our patients are healthy individuals without infections, adding systemic antibiotics to the decolonization treatment must be considered carefully. There is a risk of side effects in the individual patient, and in the era of a rising incidence of antimicrobial resistance, prudent use of antibiotics is crucial to avoid further selection of resistance [11].

As there are no published clinical trials evaluating the effect of clindamycin monotherapy for MRSA throat carriage, which has been the common practice for several years in our region, and we wish to avoid using rifampicin due to its side effects and interaction profile [12], we planned this randomized trial to fill in the gap in the literature {6b}.

### Objectives {7}

The objectives are to evaluate our current decolonization regime and test our hypothesis that decolonization treatment in combination with oral clindamycin is superior to standard treatment alone, at the second attempt of decolonization treatment.

### Outcomes {12}

The primary outcome is MRSA-negative swabs 1 month after completing the treatment.

The secondary outcome is MRSA-negative swabs 6 months after completing the treatment.

## Method and trial design {8}

This is a randomized, placebo-controlled, double-blinded trial, including patients ≥ 18 years of age who have tested MRSA-positive in their throat after completing one standard decolonization treatment. The protocol follows the SPIRIT reporting guidelines [13].

### MRSA patients

In the Capital Region of Denmark, the number of new MRSA-positive patients ≥ 18 years was 855 in 2018 with an estimated MRSA prevalence of < 0.5% [14, 15]. In around 60% of cases, MRSA is first found in a sample from an infection (mainly skin and soft tissue infections), and approximately 75% of these patients are MRSA carriers following the infection. Therefore, we expect to encounter approximately 700 newly diagnosed MRSA carriers per year of which around 66% are throat carriers in their first screening sample (462 patients). Based on retrospective data, around 40% become MRSA-free after the first decolonization treatment, which means that approximately 277 of the throat carriers ≥ 18 years old are still MRSA-positive after the first treatment. We expect that 40% of these patients have one or more exclusion criteria and therefore end up with approximately 330 patients that fulfill the inclusion criteria within the study period. As a part of our working routine, we automatically obtain information about all MRSA culture-positive samples in the lab on daily basis.

### Study settings and recruitment {9}{15}

Patients are recruited from the Capital Region via MRSA Knowledge Center Hvidovre Hospital or MRSA team, Herlev Hospital. Patients have since their first positive MRSA result an active connection to the MRSA Knowledge Center Hvidovre Hospital/MRSA team, Herlev Hospital. Patients often call for support, guidance, and treatment plans, and routine letters are often sent out to patients. When the department’s nurses or doctors have written or oral contact with an MRSA carrier that could be a possible candidate for the trial, the possibility of participating in the trial will be mentioned and the investigator’s contact details will be given.

When an eligible patient wishes to participate in the trial, oral and written consent is obtained by the investigator during a physical meeting at Hvidovre Hospital, and the participant receives a randomized decolonization package. The package includes study medicine, cleaning guide, participant information, booklet regarding rights as a study participant, and a diary to fill out daily during the study from days 1 to 14, asking about trial compliance and adverse events {32}. The investigator obtains signed informed consent (Fig. S1) and returned diaries in a secure locked room, at Hvidovre Hospital {26a}.

### Data collection plan {18a}

Participant data will be pseudonymized and will be handled according to the national rules and general data protection regulation (GDPR) {27}. A case report form (eCRF) is created for each patient for the collection of trial data using Research Electronic Data Capture (REDcap), a secure web application for managing online databases designed for non-commercial clinical research {19}. This will include the following data: project number, date of birth, age, sex, height, weight, body mass index (BMI), relevant medical history/current medication, sites and dates of MRSA-positive samples, prior MRSA decolonization treatments, household composition and household members MRSA carrier state, typing and resistance data of the MRSA strain, and laboratory and investigational results. For sexually active female participants of reproductive age, a pregnancy test is performed before enrollment, and we record the use of contraceptives. Auditing of data is performed by the GCP unit.

### Interventions {11a}

Participants are randomly allocated to receive a randomized, double-blinded {17a} decolonization package and are instructed to follow a standard 5-day cleaning regime as well as using mupirocin nasal ointment 2% and chlorhexidine body wash 4%. The treatment includes either placebo capsules to consume, 2 capsules three times daily for 10 days, or clindamycin 300-mg capsules, 2 capsules three times daily for 10 days. Placebo and clindamycin are manufactured to look exactly alike by Glostrup Pharmacy {16a/b/c}, who is also in charge of the randomization/blinding of the packages. Individual sealed and securely closed envelopes for each included participant ID have been created. An emergency unblinding procedure is established to allow the investigator the option to withdraw and unblind participants in case of an emergency {17b}.

### After the treatment

Thirty to 60 days after the end of treatment, the participants will routinely have MRSA control swabs taken by their general practitioner (GP) according to the national MRSA guidelines. Control swabs and the results of these are handled routinely. In laboratory settings, Eswabs (Copan) are inoculated in an enrichment broth containing 2.5% NaCl, 3.5 mg/L cefoxitin, and 20 mg/L aztreonam for overnight incubation at 35 °C. From the broth, 10 μL is spread on a 5% blood agar and an MRSA chrome agar (Biomérieux). Susceptibility testing is performed by disk diffusion using EUCAST breakpoints. MRSA-positive isolates are confirmed by an in-house PCR [16].

If the participant is MRSA-positive 1 month after the treatment, the participant will leave the trial, and the randomization will be unblinded in order to plan the most optimal treatment outside the trial, without revealing the given treatment to the investigator, leaving the investigator blinded until the end of the trial, approximately September 2025 (currently planned), when the final swab results from the last included participant have been analyzed (Table 1) {13}.

**Table 1:** Schedule of enrollment, interventions, and assessments

	Enrolment and allocation of treatment	Post-treatment control swab	Post-treatment control swab*
**Timepoint/assessments**	***T***_**0**_	***T***_**1 month**_	***T***_**6 month**_
Enrollment	X		
Eligibility screen	X		
Informed consent	X		
**Interventions**			
Standard decolonization + clindamycin	X	X	X
Standard decolonization + placebo	X	X	X

^*^If MRSA positive at *T*_1 month_, the participant leaves the trial and will not be sampled in the trial at *T*_6 month_

If MRSA-negative at 1 month, no unblinding will take place at this point. The participant will have new control swabs taken at the GP 6 to 8 months after completing the treatment, as generally recommended. If MRSA-negative at 6–8 months after treatment, the participant is declared MRSA-free according to the national MRSA guidelines.

### Sample size {14}

The anticipation is a 30% increased decolonization rate based on non-published data described in the background and rationale section in the group receiving standard treatment plus clindamycin compared with the group treated with standard treatment and placebo.

Power calculation shows that we will need 31 patients in each group. It is our goal to include 40 patients in each group, as we expect that 9 patients in each group will either drop out or be non-compliant to the treatment.

### Compliance and adherence {11c}

Compliance is defined by having consumed more than 80% of the oral capsules. We expect patients in both groups to be equally compliant. In the diary handed out with the decolonization package, study subjects are instructed to record how many capsules, if any, are left after the treatment to monitor compliance. The diary should be returned as quickly as possible to the investigator, with any leftover capsules in a pre-stamped envelope. The number of returned capsules will be reported in the eCFR. If participant control swab results are not registered approx. 30 days after completing the treatment, or diary and left-over capsules are not returned, friendly reminders are sent to the participant by email {18b}.

### Eligibility criteria {10}

Inclusion criteria:

- Age ≥ 18 years
- MRSA carriage in the throat after the first topical decolonization treatment (regardless of previous swab results)
- Has completed one standard topical decolonization treatment

Exclusion criteria:

- Pregnant or lactating woman
- Sexually active women of reproductive age that do not use approved contraceptives
- Cannot read or speak Danish
- Skin infection or other active infections
- Activity in skin diseases such as eczema or psoriasis
- MRSA isolate resistant to clindamycin (defined by inhibition zone size < 22 mm using EUCAST disk diffusion methodology) or resistant to mupirocin
- Allergy to clindamycin, chlorhexidine, or mupirocin
- Taking medications that interact with clindamycin according to the medicine information leaflet
- MRSA-active antibiotic treatment within 7 days before inclusion in the study or during the study period
- Followed by specialist due to liver disease
- Severe overweight (body mass index (BMI) > 35) or weight < 50 kg
- Indwelling percutaneous permanent devices such as intravenous catheters or urinary tract catheters
- Daily contact with pigs or minks (decolonization is generally not offered, according to the National Board of Health)
- Nursing home resident or health care worker (alternative swab regime is recommended)
- Not being capable of completing another treatment successfully
- MRSA-positive household members younger than 2 years (MRSA-positive children below 2 years of age and their household members are generally not offered decolonization treatment according to the National Board of Health)
- MRSA-positive household members, where it is determined that further decolonization attempts are not indicated

### Modifications {11b}

Participants will be informed that participation is voluntary and that they have the right to leave the study at any given time, without providing a reason, and with no loss of benefits concerning treatment outside the study. If a participant withdraws, we will be able to use the results that we have at the given time of the withdrawal, as mentioned in the handout information. The investigator has the right to terminate participation if considered in the best interest of a given participant.

### Concomitant care {11d}

Hospitalization and other interventions interfering with the compliance of the trial will inevitably result in terminated trial participation.

### Disadvantages, advantages, risks, and adverse events

The disadvantages include antibiotic consumption by healthy individuals. It is well-known that antibiotic consumption changes the microbiome of the intestine for a longer period, but the impact of this influence is still rather unknown. Furthermore, antibiotic consumption leads to selection for antibiotic resistance. According to the investigational product information, less than 10% of clindamycin consumers will experience side effects, most commonly of gastrointestinal characters such as abdominal pain and diarrhea. Skin rash is also a common side effect seen in 1 to 10%.

Advantages for the participant receiving clindamycin include the possibility of becoming MRSA-free at an earlier time point, than had the carrier not participated in the trial.

The greatest advantage is gathering evidence to optimize the treatment of future MRSA carriers.

The results from this study will impact the future recommendations for decolonization regimens.

### Harms {22}

An adverse event (AE) is any untoward medical occurrence in a study subject administered a test product, whether considered related to the study treatment or not.

AEs recorded in the diary, including onset, end, intensity, causality, action taken, and outcome of the AE, will be reported in the eCRF (REDCap). Participants will be followed up by the medical judgment of the investigator, until the abnormal condition is resolved or the investigator deems further observations or examinations to be no longer medically indicated.

Causality is evaluated by the investigator and sponsor. In case of incongruency, both evaluations will be reported.

#### Serious adverse events

A serious adverse event (SAE) is any untoward medical occurrence that, at any dose, is life-threatening at the time of the event, requires hospitalization or prolongation of existing hospitalization, results in persistent or significant disability or incapacity, is another important medical event, results in congenital anomaly or birth defect, or results in death. If an SAE occurs, the SAE will be reported to the authorities and will be handled in accordance with local regulations.

Known adverse reactions of clindamycin may be experienced in approximately 0.1–10% according to the clindamycin product information, most commonly:

- Gastrointestinal: abdominal pain, pseudomembranous colitis, nausea, vomiting, and diarrhea
- Hypersensitivity reactions: generalized mild to moderate morbilliform-like (maculopapular) skin rash
- Liver: jaundice and abnormalities in liver function tests have been observed during clindamycin therapy

### Statistics {20a/b/c}

Continuous data are expressed as median and range. Power calculations are based on an expected proportion of 20.3% in the control group and a minimum relevant difference of 30% points resulting in an expected 50.3% in the intervention group. Power is set at 80 and significance level at 0.05%. Since only superiority is of interest, a one-tailed *p*-value will be used. The statistical software “R” [17] was used for power calculation. The analysis will be performed using Fisher’s exact test or chi-squared test depending on sample size. Statistical calculations will be performed using IBM SPSS Statistics, version 25, IBM Corporation, and other(s) 1989, 2018. We intend to include every study participant randomized in the primary analysis, only omitting excluded participants, where no data is available after randomization or in case of lack of compliance. Missing data will be excluded from the analysis. No subgroup or interim {21b} analysis is planned.

## Discussion

If the bacterial isolate is susceptible, clindamycin is often an excellent treatment choice for MRSA infections [18]. In Denmark, to keep MRSA prevalence low, asymptomatic MRSA carriers are offered decolonization treatment, but several decolonization attempts are often required to obtain permanent decolonization, and clindamycin is frequently added to the third decolonization treatment in the anticipation that the addition of clindamycin increases treatment success.

Previously, a few studies have indicated that MRSA decolonization is more successful when antibiotics are added including a Swedish randomized, controlled study, based on 52 patients, investigating the effect of rifampicin in combination with either clindamycin or sulfamethoxazole/trimethoprim [10], and two other retrospective studies on 293 Swedish children < 18 years [19] and 177 Dutch patients between 12 and 73 years [20], respectively. Other retrospective studies were unable to find statistical significance of added systemic antibiotics [5, 6]. Due to the lack of randomization in most of these studies, evidence is low, and no randomized study has examined the effect of clindamycin alone, which is the current preference in our region. As hundreds of healthy MRSA carriers have been treated with clindamycin in the last decade the clinical relevance of this trial is high. Clindamycin consumption has a price in terms of cost, possible side effects, and possible selection of antimicrobial resistance [21]. Therefore, it is crucial to determine if the benefit of oral clindamycin consumption for decolonizing asymptomatic MRSA carriage is worth the price.

This prospective, randomized, placebo-controlled, double-blinded trial will fill in the gap in the literature and help us determine whether clindamycin should have a place in MRSA decolonization.

### Ancillary and post-trial care {30}

Participants in the trial will not receive any form of financial compensation but are covered by the national patient insurance according to “Lov om klage- og erstatningsadgang inden for sundhedsvæsenet” (The Danish Ministry of Health, 2017).

### Data access {29}

The information obtained in the trial will be available for the sponsor, investigator, the Regional Research Ethical Committee, the Danish Medicines Agency, and the GCP unit. Anonymous stored data will be available on reasonable request after all analyses have been completed {31c}.

### Dissemination policy {31a}

The results will be published as quickly as possible, in an anonymous form, in a form where participants cannot be identified. The reporting of the study results will follow the CONSORT 2010 statement [22]. The publication will be in the form of a scientific article, in an internationally recognized peer-reviewed journal. Both negative, inconclusive, and positive results will be published. Data will be kept 5 years after termination of the trial and will then be destroyed.

Study participants can check a box on the “written consent,” if they would like to learn more about the study results, which will be sent by email once finalized.

## Trial status

Recruitment of study participants began on May 28, 2020, and is currently planned until we reach the desired number of participants, estimated before September 2025.

## Supplementary Information

**Additional file 1.** Informed consent form, to be signed by participant and investigator.

## Abbreviations

MRSA: Methicillin-resistant *Staphylococcus aureus*
GP: General practitioner
BMI: Body mass index
eCRF: Electronic case report form
AE: Adverse events
SAE: Serious adverse events
GCP: Good Clinical Practice
GDPR: General data protection regulation
RCT: Randomized controlled trial
