# Supplementary figures and images for: A randomized, placebo-controlled, double-blinded trial of MRSA throat carriage treatment, with either standard decolonization alone or in combination with oral clindamycin
Source: Trials. 2022 Jun 16;23:502. doi: 10.1186/s13063-022-06443-1 (PMC9205106; doi:10.1186/s13063-022-06443-1)

**Supplementary Figure 1**

Informed consent form, to be signed by participant and investigator.


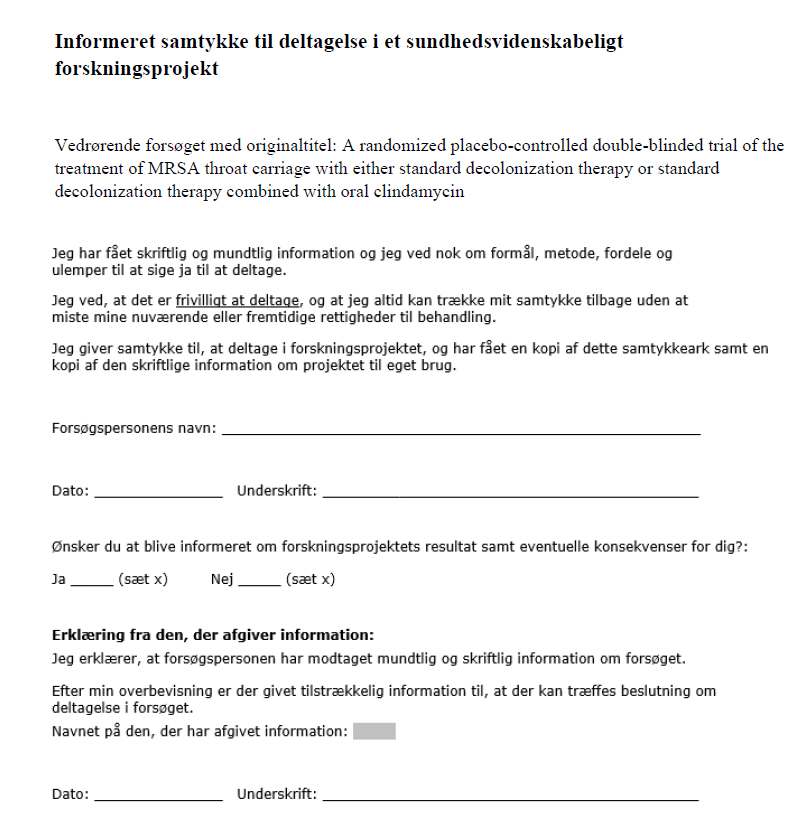

Supplement: Supplementary file 1 — Additional file 1. Informed consent form, to be signed by participant and investigator. [file 13063_2022_6443_MOESM1_ESM.docx]
